# The resonance® metallic ureteric stent in the treatment of chronic ureteric obstruction: a safety and efficacy analysis from a contemporary clinical series

**DOI:** 10.1186/s12894-017-0204-8

**Published:** 2017-03-10

**Authors:** C. Patel, D. Loughran, R. Jones, M. Abdulmajed, I. Shergill

**Affiliations:** 1 Department of Urology, Wrexham Maelor Hospital, Croesnewydd Rd, Wrexham, LL13 7TD Wales; 2 North Wales and North West Urological Research Centre, Croesnewydd Rd, Wrexham, LL13 7TD Wales

## Abstract

**Background:**

We evaluate the efficacy and safety of metallic ureteric stenting using the Cook Resonance® stent in the treatment of chronic ureteric obstruction of benign and malignant aetiology. Published experience of using this stent in this context is limited. We add to the body of literature on this topic.

**Methods:**

All patients who had a Resonance® metallic stent inserted between April 2009 and November 2014 in our institution were identified from a prospectively maintained stent-database. Primary outcome was relief of ureteric obstruction, defined by successful clinical and radiological treatment of hydronephrosis/hydroureter. Secondary outcome measures included operative time, radiological exposure, total stent dwell time (defined as the cumulative time in months for which a Resonance® metallic stent was in situ), and early and late complications.

**Results:**

Twenty-one patients underwent 52 stent insertion episodes (SIE). Median age was 58 years (range 39–90). Stent insertion resulted in successful treatment of hydronephrosis/hydroureter in 96% (2 SIE resulted in failure to relieve ureteric obstruction). Median operative time was 21 min (range 12–90) Median radiation exposure was 815.3 cGy/cm2 (range 192.9–5366.3). Median stent dwell time was 19.5 months (range 6–52) in non-malignant and 12 months (range 2–48) in malignant ureteric obstruction. One stent migrated proximally during insertion and had to be retrieved using an antegrade approach. 5 patients re-admitted with haematuria: all resolved without intervention or blood transfusion. 3 episodes of post-operative urinary infection were recorded; all were successfully treated with oral antibiotics.

**Conclusion:**

Metallic ureteric stenting using the Resonance® stent is safe and effective for treating ureteric obstruction from both malignant and benign causes. The success rate in our series is 96%.

## Background

Ensuring adequate long term renal drainage in the context of chronic ureteric obstruction can be a challenge for the urologist. Traditionally, the two main options available for treatment are indwelling polymeric ureteral stents (polyurethane, silicone or hydrogel) and percutaneous nephrostomies. Percutaneous nephrostomy is more invasive and often susceptible to tube blockage and dislodgement and a reduced quality of life. Indwelling-stent failure rates in extrinsic compression are reported at 36–58% [1–5]. They are also commonly associated with device encrustation (thereby requiring bi-annual exchange), biofilm development and subsequent urinary tract infection, and reduced intra-luminal flow in the context of extrinsic compression [6].

The all-metal Cook Resonance® stent has been shown to have much better intraluminal flow compared to polymeric stents (5.15 vs. 0.64 mls/min) [6] and reduced stent encrustation (requiring annual exchange only and more cost effective than standard polymer stenting [7]). The improved intraluminal flow is explained by the Resonance® stent’s spiral coil design resulting in bending of the coils as opposed to buckling, which requires much higher external force to cause compression (it maintains 50% of its internal diameter with 31 pounds of compressive force placed at its’ proximal, middle and distal segments [8]). The occluded end design means urine drains into the lumen of the stent through small gaps between the spirally wound coils. Stent encrustation led to polymer stent manufacturers advising bi-annual stent changes. Due to reduced stent encrustation, the Resonance® stent requires annual exchange only.

Published experience with the Cook Resonance® stent to date is limited and includes case series’ with small numbers of patients [9–19]. The aim of our study was to evaluate the efficacy and safety of the Resonance® stent in our experience in the treatment of chronic ureteric obstruction from both benign and malignant causes, and add to the body of literature on this topic.

## Methods

From our prospectively maintained electronic stent database, we identified all patients in whom a Cook Resonance® metallic stent was inserted between April 2009 and Nov 2014. In total 21 patients underwent 52 stent insertion episodes (SIE) during this study. Follow up data was obtained from detailed review of patient case-notes and electronic records.

The primary outcome measure was relief of ureteric obstruction as defined by successful clinical and radiological treatment of hydronephrosis +/− hydroureter. Treatment failure was also defined as removal of the stent before the recommended removal date. Secondary outcome measures included surgical operative time, radiological exposure during fluoroscopy, total *stent dwell time* (defined as the cumulative time period in months for which a Cook Resonance® metallic stent was in situ) as well as early and late post-procedural complications, defined using the modified Clavien-Dindo classification [20].

All patients had previously had their ureteric obstruction managed by either Double J® polymer stenting or nephrostomy. The decision to change to a Cook Resonance® metallic stent was consultant led and based on the patient’s individual experience with previous stent/nephrostomy, clinical indication, life expectancy and patient preference.

Cook Resonance® metallic stent was performed as previously described [21]. Briefly, under general anaesthesia, a guidewire was inserted using a combination of cystoscopic and fluoroscopic guidance, followed by a coaxial introduction of the Resonance® sheath and catheter, which contains a radio-opaque tip. The guidewire and catheter were then exchanged for the closed ended Cook Resonance® stent through the sheath, with the aid of the catheter as a pusher. The Resonance® sheath is then finally removed to leave the stent in position. The length of the stent used was based on published literature [22].

Follow up included urine microscopy and imaging (ultrasound or CT depending on clinical indication), and all hospital visits in the post-operative period were recorded. Routine stent exchanges due at 12 months were triggered by the electronic stent register.

## Results

Of the patients 7 were male and 14 female. Median age was 58 years (range 39 to 90). Of all the SIE, 35 (67%) had a benign cause (see Table 1). All patients had 6Fr stents of varying lengths (Table 2). Median stent duration was 12 months (range 0.5–14). Median clinical follow up was 12 months (range 2–52) (Table 3).

**Table 1:** Stent Insertion Episode (SIE) by underlying cause

Underlying cause of extrinsic ureteric compression	Associated Number of SIE
Retroperitoneal fibrosis	24
Neuropathic bladder	4
PUJ stricture	3
Duplex kidney	2
Obstructed transplant kidney	2
Malignancy (direct or nodal compression on ureter)	17

**Table 2:** Stent length distribution for SIE

Resonance Stent length (cm)	Number of SIE
26	14
24	23
22	11
18	2
14	1
12	1

**Table 3:** Number of stent exchanges analysed by patient

Total number of Stent Exchanges	Number of patients
0 (i.e., removed/deceased ≤ 12 months)	13 (9 deceased, 4 removed)
1	5
2	1
3	2

### Primary outcome results

The stent failure rate in our experience was 4%–; 2 SIE resulted in failure to successfully relieve hydronephrosis +/− hydroureter. Success rate of initial retrograde insertion was 98%, with no intraoperative complications. 1 stent had to be inserted in an antegrade fashion after percutaneous renal access.

### Secondary outcome results

Median surgical time was 21 min (range 12–90) (this included 15 bilateral stent insertions at the same procedure). Median radiation exposure (measured as kerma-air-product, P_ka_) was 815.3 cGy/cm^2^ (range 192.9–5366.3).

Success rate of initial retrograde insertion was 98%, with no intraoperative complications. 1 stent had to be inserted in an antegrade fashion after percutaneous renal access.

Median stent duration was 12 months (range 2–52). However, when sub-categorised into benign versus malignant aetiology, median stent dwell time (cumulative time period in months for which a Resonance® stent was in-situ) was 19.5 months (range 6–52) for the former and 12 months (range 2–48) for the latter (in all non-deceased patients). 10 (47%) patients died during the follow up period with a metallic stent in-situ. The median stent dwell time in these patients was 7.5 months (range 0.5–14).

Urine microscopy was performed within the first 14 days post-operatively in thirty-eight (73%) of fifty-two stent episodes. Thirty-five (92%) showed no infection, two cultured *E. coli* (both on the same patient with poorly-controlled diabetes) and one grew *Pseudomonas aeruginosa* (patient with an indwelling catheter). These required oral antibiotic treatment only (grade I complication according to the modified Clavien classification system). Haematuria requiring hospital admission occurred in five (24%) patients. All were managed conservatively without the need for intervention or transfusion (grade I). One stent had to be retrieved through an antegrade approach due to incorrect placement of the distal end into the ureter at the time of retrograde insertion (grade III-a). Removal of stents (bilateral in situ) was performed in one patient due to significant pain (grade III-b). All the remaining stent episodes (96%) successfully relieved hydronephrosis +/− hydroureter on subsequent imaging (ultrasound or CT). 9 patients died within 12 months of stent insertion. Malignancy accounted for 5 (56%) of these and underlying medical co-morbidities for the remainder.

**Fig. 1:**
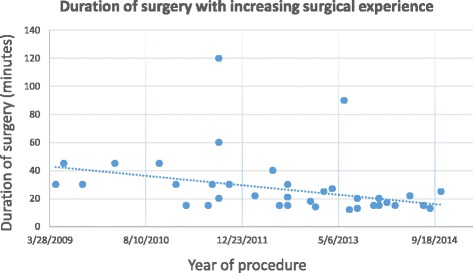
Duration of surgery with increasing experience of Cook Resonance® stent insertion

**Fig. 2:**
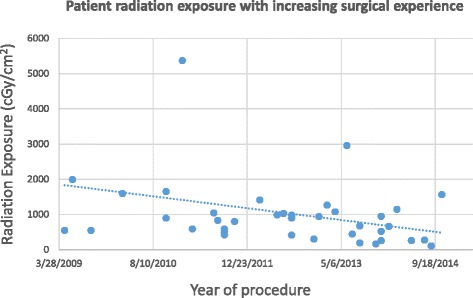
Radiation exposure to the patient with increasing surgical experience

Duration of surgery and radiation exposure to the patient were both seen to reduce as more experience was gained of performing the procedure (Figs. 1 and 2).

## Discussion

Since its’ introduction in 2006, the Cook Resonance® metallic ureteric stent has gained increasing popularity as an option for relieving ureteric obstruction from malignant and benign pathologies. It is the design that lends itself to this purpose. This case series contributes significantly to the limited literature published on the safety, efficacy and tolerability of this stent in clinical practice.

Retrograde insertion was achieved for the closed-ended Resonance® stent (using a coaxial technique of sheath and introduction catheter) in 51 of 52 episodes. Antegrade insertion was performed in 1 episode due to failed retrograde insertion from inability to pass a guidewire past the distal ureter. Liatsikos et al. [9] have previously chosen the antegrade approach in patients with long strictures or lower ureteric strictures. They safely used balloon-dilatation over an antegradely inserted standard 0.0035 in. guidewire to allow passage of the 8.3Fr outer introducer sheath. Patient selection to identify potentially difficult retrograde insertion would avoid a general anaesthetic in a high risk patient. ‘Stent failure’ as defined by failure of decompression of hydronephrosis/hydroureter occurred in only 2 (4%) of stent episodes. This was despite uncomplicated retrograde insertion and as a result of the individuals’ underlying malignant disease. Published figures for stent failure from case series’ of greater than fifteen patients vary from 16–35% [7, 9, 10, 18]. This wide variation can be explained by the different study sample sizes, which on the whole remains relatively low. The largest series to date only had 50 patients and reported a 16% stent failure rate, interestingly all with a non-cancerous cause of renal or ureteric obstruction [9]. We believe our low stent failure rate was due to case selection in our series, rather than all comers.

Cook advise stent exchange at 12 months. This is therefore our departmental policy. Overall median stent duration was noted as being 12 months (range 0.5–14). Due to our long follow-up period (upto 52 months), some patients had multiple stent exchanges. When this was factored in to the analysis of sub-categorisation by aetiology, median *stent dwell time* (cumulative time period in months for which a Resonance® stent was in-situ) was longer in benign compared to malignant cases. This difference can be explained by the higher mortality rate amongst the group with an underlying malignancy as a cause of ureteric obstruction. To our knowledge, this series has the longest follow-up duration to date. Stent patency at 14 months has previously been reported [9] and therefore raises the potential for a longer time duration between exchanges.

Radiation exposure associated with this procedure was low at a median of 815 cGy/cm2. A significant dose (requiring patient follow-up) for interventional radiology procedures is defined as a kerma-air-product of greater than 50,000 cGy/cm2 [23]. Furthermore, both radiation exposure to the patient and procedure duration reduced in a linear fashion from 2009 to 2014.

The overall majority of complications were grade I according to the modified Clavien classification system. Urinary tract infection (UTI) within the first 2 weeks post-stenting occurred in 3 (8%) cases, twice in the same patient but after different stent insertion episodes. Underlying co-morbidities are likely to have increased this risk (poorly controlled diabetes and an indwelling catheter). The British Association of Urological Surgeons quote a UTI rate of 2–10% post cystoscopy and ureteric stenting. Our figures for the Resonance® metallic stent are comparable but on a very limited study size and in patients with risk factors for infection. Haematuria requiring hospital admission was seen in 5 (25%) patients. They were all managed conservatively. It can be argued that counselling for primary care to better inform them on post-stenting care could reduce unnecessary, and costly, hospital admission. Similarly it is possible that some patients may have experienced transient haematuria but not reported it. Previous studies with greater than 15 patients have reported haematuria in 6–21% of patients [9, 14].

Pain has not been identified as a major issue, with only one patient requiring stent removal (bilateral stents) as a result.

Nine patients died within 12 months of stent insertion. Malignancy accounted for greater than half of these and all deaths were secondary to the underlying disease process. As can be expected the median *stent dwell time* was higher in benign compared to malignant cases.

Sample size can be viewed as a limitation of our study. Whereas our database continues to expand, this is at a relatively slow rate given that metallic stenting is still on the whole reserved for a select group of patients. We hope that changing clinicians’ thinking through this critical analysis of the safety, efficacy and tolerability of this stent will lead to a larger number of patients being made eligible.

A further limitation is that we did not perform a cost analysis to compare the Resonance® stent with the standard polymeric stent. The convenience to the patient is an annual as opposed to bi-annual stent exchange. Only one study to date has performed a cost analysis [7]. In it Lopez-Huertas et al. reported a significant (43%) cost saving with the Resonance® metallic stent compared to the Percuflex™ stent in a thirteen patient cohort.

## Conclusion

We conclude that the Resonance® metallic stent is safe and effective for treating ureteric obstruction from both malignant and benign causes. Increased awareness of this potential will allow more patients to benefit from an annual (as opposed to bi-annual) stenting procedure.

## Abbreviations

CGY: Centi-gray
CT: Computed tomography
Fr: French
Pka: Kerma-air-product
PUJ: Pelvi-ureteric junction
SIE: Stent insertion episodes
